# Functionalization of Amorphous and Crystalline Calcium Phosphate Nanoparticles with Urea for Phosphorus and Nitrogen Fertilizer Applications

**DOI:** 10.1021/acs.jafc.5c03970

**Published:** 2025-06-30

**Authors:** Salem Ghribi, Lorenzo Degli Esposti, Blaire Steven, Nubia Zuverza-Mena, Jing Yuan, Jacquelyn C. LaReau, Jason C. White, Deb P. Jaisi, Alessio Adamiano, Michele Iafisco

**Affiliations:** † Institute of Science, Technology and Sustainability for Ceramics (ISMMC), National Research Council (CNR), 48018 Faenza (RA), Italy; ‡ Department of Mathematical, Physical and Computer Sciences, University of Parma, 43124 Parma, Italy; § Dipartimento di Chimica e Chimica Industriale, Università degli Studi di Genova, 16146 Genova, Italy; ∥ Department of Environmental Science and Forestry, 5758 The Connecticut Agricultural Experiment Station, New Haven, Connecticut 06511, United States; ⊥ Department of Analytical Chemistry, The Connecticut Agricultural Experiment Station, New Haven, Connecticut 06511, United States; # Interdisciplinary Science and Engineering Laboratory, Department of Plant and Soil Sciences, 5972 University of Delaware, Newark, Delaware 19716, United States; □ 5758 The Connecticut Agricultural Experiment Station, New Haven, Connecticut 06511, United States

**Keywords:** amorphous calcium phosphate, hydroxyapatite, nanoparticles, urea, controlled-release fertilizers

## Abstract

The development of nanofertilizers has gained significant attention for their potential to enhance nutrient delivery to plants while mitigating the environmental impact of intensive agriculture. In this study, we investigated the urea functionalization of two types of calcium phosphate nanoparticles, hydroxyapatite (HAP) and amorphous calcium phosphate (ACP), as nanofertilizers capable of simultaneously releasing phosphorus and nitrogen in a controlled manner. Leaching experiments using a vermiculite column revealed that ACP-urea significantly slowed urea release compared with free urea, whereas HAP-urea exhibited a release profile similar to that of free urea. Greenhouse experiments on corn (*Zea mays*) showed that ACP-urea treatment enhanced the dry biomass and relative chlorophyll content compared to treatments with free urea combined with either ACP or monocalcium phosphate. Microbiome analyses indicated that the improved plant performance with ACP-urea was primarily due to the material’s physicochemical properties rather than significant shifts in the rhizosphere bacterial communities. These findings highlight the potential of ACP nanoparticles as effective nanofertilizers for controlled phosphorus and nitrogen release, contributing to more sustainable agricultural practices.

## Graphical abstract

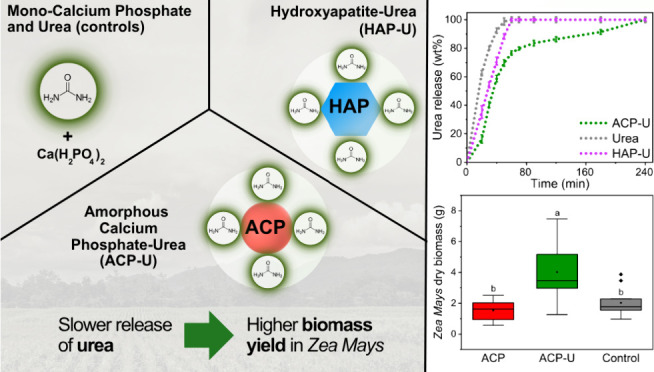

## 1 Introduction

The global commitment to sustainable farming, as outlined in the United Nations 2030 Agenda for Sustainable Development, has widely promoted the development of innovative strategies to address the dual challenge of feeding a growing population while using natural resources responsibly.^1^ A key priority in this effort is to significantly improve crop yields despite the decreasing farmland and new regulations constraining or limiting the use of land for agriculture.

Nitrogen and phosphorus are critical macronutrients essential for plant growth. However, due to the inefficiencies in current fertilizing practices, only a fraction of the applied phosphorus and nitrogen is taken up by plants, and the excess is either immobilized in the soil or lost to the environment, leading to both financial losses and harm to the environment.^2,3^ A critical metric to assess fertilizer effectiveness is nutrient use efficiency (NUE), a measure of plants’ ability to accumulate and metabolize nutrients in plant tissues. Currently, conventional nitrogen-based fertilizers typically exhibit NUE values below 50%,^4^ indicating that over half of the nitrogen supplied is lost to the environment. Similarly, with phosphate fertilizers, only a small proportion (about 30%) of the provided phosphorus is acquired by crops.^5^ These limitations point to a pressing need to develop novel, more efficient fertilizers to minimize nutrient losses and their adverse environmental impacts. To address this challenge, recent attention has turned to tapping nanotechnology potential to revolutionize plant nutrition and mitigate environmental impacts.^6^ Due to their unique properties, including controlled solubility and larger surface area, nanomaterials offer opportunities for controlled nutrient release and precision targeting, allowing for increased nutrient loading.^7^ Developing innovative fertilizer formulations, particularly those that leverage nanotechnology to enhance the delivery of nutrients to crops while minimizing the environmental loss, presents a promising avenue for intensifying sustainable agricultural productivity.

Among nanomaterials, calcium phosphates (CaPs) in the form of crystalline hydroxyapatite (HAP) or amorphous calcium phosphate (ACP) show great promise in agriculture as sustainable fertilizers due to their controlled nutrient release and high bioavailability.^8^ A pioneering study by Liu et al.^9^ introduced HAP nanoparticles as a highly effective fertilizer with significant agronomic advantages. This research demonstrated that applying HAP nanoparticles led to a 33% increase in the soybean growth rate and a 20% increase in seed yield compared to dihydrogen calcium phosphate (Ca­(H_2_PO_4_)_2_) as a conventional phosphorus fertilizer.^9^ Similarly, in a greenhouse study on lettuce (*Lactuca sativa*), ACP has been shown to enhance crop yields by 20% compared to the conventional phosphorus fertilizer.^10^

CaP nanoparticles can offer an optimal balance between traditional phosphorus sources and phosphate rocks. The latter releases nutrients too slowly, often failing to meet plant needs, while traditional soluble phosphorus salts can lead to environmental issues such as nutrient leaching/runoff and water eutrophication. In contrast, CaP nanoparticles provide a controlled and sustained release of phosphorus, minimizing the environmental impact while meeting plant demand, making it a promising advancement in sustainable agriculture.^9,11^ CaPs are also notable for their pH-responsive solubility (higher solubility at acidic pH), enabling the controlled release of calcium and phosphate ions in response to soil pH fluctuations. This gradual and regulated nutrient release could be synchronized with crop growth stages, reducing nutrient losses through leaching and thus minimizing waste. Enhanced nutrient uptake efficiency contributes to the major goal of sustainable agricultural practices in a changing climate.^12^

CaP-based nanomaterials can be synthesized through various methods or derived from natural sources, such as bone wastes.^13^ Among synthetic approaches, the wet precipitation method is the most used and widely favored in the literature due to its simple design and absence of organic solvents, merged with its reproducibility, scalability, and tunability of particle size and morphology. Other methods, such as hydrothermal and solid-state processes, are less frequently used but can offer distinct advantages depending on the application.^12^ Nanostructured CaPs have also been utilized as carriers for both micro-^14^ and macronutrients.^15^ Their high surface area enables loading of various ions and cations, making them a versatile nutrient delivery system.

Urea is the most common and widely used nitrogen fertilizer across the globe in agriculture, promoting improved plant growth and yield.^16^ Urea can be incorporated into CaPs using two primary approaches: the postsynthetic method, in which urea is added to preformed CaP nanoparticles,^17^ and the one-pot synthesis approach, where urea and CaP are coprecipitated simultaneously to achieve homogeneous incorporation.^18^

Kottegoda et al. demonstrated that HAP functionalized with urea via one-pot synthesis can effectively slow nitrogen release, aligning nutrient availability with plant needs. This property enhances NUE by synchronizing nutrient release with plant uptake.^19^ Similarly, Maghsoodi et al. showed that HAP-urea nanorods synthesized via a one-pot method significantly reduced the release rate of urea in water, slowing its dissolution by a factor of 11.5 over 460 s compared to that of pure urea. In addition to regulating urea release, these materials enhanced nitrogen retention by slowing the conversion of urea to ammonium, helping to maintain more stable nitrogen levels in the soil over extended periods.^20^ The controlled nutrient release properties of these materials not only meet plant demands but also support more sustainable nutrient management in agriculture.^15,19,21^ Ramìrez-Rodrìguez et al. reported the development of ACP nanoparticles loaded with urea through a one-pot synthesis. Their findings showed that maize plants achieved comparable growth yields using 40% less nitrogen.^18^ Additionally, Carmona et al. demonstrated that postsynthetic modification techniques could triple the nitrogen-loading capacity of ACP materials. When applied to hydroponic cucumber (*Cucumis sativus*) plant growth, these materials achieved growth rates comparable to those of conventional methods despite a reduced nitrogen application rate.^17^

The aim of the current study is to functionalize two types of CaP nanoparticles, namely, crystalline HAP and amorphous ACP, with urea, and to assess their potential as controlled release fertilizers. The urea loading capacity of the nanoparticles was evaluated, and the functionalized samples were thoroughly characterized. Urea release was investigated in a vertical bed column packed with vermiculite. Based on its ability to slow down urea release with respect to HAP-urea and free urea, the ACP-urea sample was further tested in a greenhouse experiment with corn to assess its potential to enhance plant growth relative to conventional fertilizers. The rhizosphere microbial community of the soil was also analyzed to determine potential impacts on the plant-associated microbiome. This study provides valuable insights into the potential of urea-loaded ACP and HAP as effective, environmentally friendly nanofertilizers.

## 2 Materials and Methods

### 2.1 Materials

All solutions for the synthesis and characterization of the materials were prepared with ultrapure water (>18.2 MΩ·cm at 25 °C) obtained with an Arium pro purification system (Sartorius, Goettingen, Germany). The following chemicals were used: calcium chloride dihydrate (CaCl_2_·2H_2_O, ≥99.0% pure), hydrochloric acid (HCl, ≥37.0% pure), sodium citrate tribasic dihydrate (Na_3_(C_6_H_5_O_7_)·2H_2_O, ≥99.0% pure), sodium phosphate dibasic dihydrate (Na_2_HPO_4_·2H_2_O, ≥99.0% pure), sodium carbonate monohydrate (Na_2_CO_3_·2H_2_O, ≥99.0% pure), calcium acetate (Ca­(CH_3_COO)_2_, 99.0% pure), urea (CH_4_N_2_O, ≥99.0% pure), potassium chloride (KCl, >99.0% pure), phosphoric acid (H_3_PO_4_, 85.0% pure), nitric acid (HNO_3_, 68.0% pure), monoammonium phosphate (NH_4_H_2_PO_4,_ ≥99.0% pure, referred to hereafter as MAP), aqueous ammonia (NH_4_OH, 28.0% pure), urea (CH_4_N_2_O, >99% pure), acetonitrile (C_2_H_3_N, >99.0% pure), vermiculite type IV, p-dimethylaminobenzaldehyde (C_9_H_11_NO, 99.0% pure, referred to hereafter as DMAB), and monocalcium phosphate (Ca­(H_2_PO_4_)_2_, >99.0% pure, referred to hereafter as MCP). All chemicals were purchased from Sigma-Aldrich (St. Louis, MO, USA).

### 2.2 Synthesis of ACP, ACP-U, HAP, and HAP-U Nanoparticles

ACP was synthesized as reported by Iafisco et al.^22^ Two aqueous solutions (500 mL each) were prepared. Solution A contained CaCl_2_·2H_2_O (0.1 M) and Na_3_(C_6_H_5_O_7_)·2H_2_O (0.1 M), while solution B contained Na_2_HPO_4_·2H_2_O (0.12 M) and Na_2_CO_3_·2H_2_O (0.2 M). Solutions A and B were combined at ambient temperature, resulting in the instantaneous precipitation of the ACP particles. The precipitated particles were separated from the supernatant by centrifugation at 15,300 × *g* (10,000 rpm) for 3 min at 4 °C using a Sorvall Legend XTR centrifuge equipped with an F15-6x100Y fixed-angle rotor (Thermo-Fisher, Waltham, MA, USA). The particles were washed three times with ultrapure water and resuspended in water at a final concentration of 10 mg mL^–1^. HAP nanoparticles were synthesized using the method described by Sandhofer et al.^23^ A 500 mL solution of H_3_PO_4_ (0.21 M) was gradually added dropwise to a 500 mL solution of Ca­(CH_3_COO)_2_ (0.35 M) at room temperature while maintaining a constant pH of 10 by adding NH_4_OH. The reaction mixture was stirred overnight at room temperature and then left still for 2 h. The nanoparticles were recovered by centrifugation, as reported above. The recovered particles were washed several times with ultrapure water and resuspended at a final concentration of 10 mg mL^–1^. ACP and HAP nanoparticles were functionalized with urea by adsorption. A 10 mg mL^–1^ urea solution was added to ACP and HA suspensions (10 mg mL^–1^) in a 1:1 proportion so that the weight of urea was equal to the weight of ACP/HA in each suspension. The mixture was stirred at room temperature for 2.5 h, following the method described by Kottegoda et al.^19^ The resulting materials, named ACP-U and HAP-U, were isolated by centrifugation as reported above, washed with ultrapure water several times, and freeze-dried.

### 2.3 Materials Characterization

The powder X-ray diffraction (PXRD) patterns of the samples were recorded by using a D8 Advance diffractometer (Bruker, Karlsruhe, Germany) equipped with a Lynx-eye position-sensitive detector. The instrument utilized Cu Kα radiation (λ = 1.54178 Å) generated at 40 kV and 40 mA. Spectra were collected over a 2θ range of 15 to 60°, with a step size of 0.02° and a counting time of 0.5 s per step.

Fourier-transform infrared (FT-IR) spectroscopic analyses were performed using a Nicolet iS5 spectrometer equipped with an iD7 attenuated total reflectance (ATR) accessory (Thermo Fisher, Waltham, MA, USA). Measurements were taken with a resolution of 2 cm^–1^, accumulating 64 scans over a spectral range of 4000 to 400 cm^–1^. Spectra deconvolution in the range between 1300 to 1800 cm^–1^ was carried out using the MagicPlot software (version 2.5.1) and considering each contribution as a Gaussian curve.

Calcium and phosphorus quantification was carried out by inductively coupled plasma–optical emission spectrometry (ICP–OES) using an Agilent 5100 spectrometer (Agilent Technologies, Santa Clara, CA, USA). For the analysis of solid samples, 10 mg of each sample was weighed and dissolved in 50 mL of a 2.0 wt % HNO_3_ solution. For the analysis of liquid samples, 4 mL of the sample was analyzed after being acidified with 150 μL of HNO_3_ (68 wt %), obtaining a final concentration of 2.0 wt % of acid.

The nitrogen content in dry ACP-U and HAP-U was measured using a FlashSmart NC soil elemental analyzer (Thermo Fisher Scientific, Waltham, MA, USA), and the results were used to estimate the urea content. Approximately 10 mg of each sample was analyzed in triplicate.

Urea quantification in aqueous solution was performed following the method described by Giraldo and Rivas.^25^ This colorimetric method was first applied to quantify urea in solid samples by dissolving them in a 3.7 wt % HCl solution. The results obtained by this method were then compared with those from CHN analysis to confirm the reliability of the quantification. Briefly, for the colorimetric analysis, a 20 mmol L^–1^ solution of DMAB in acetonitrile was prepared. Subsequently, 1.8 mL of this solution was mixed with 1 mL of the analyte solution and 37 μL of concentrated HCl (37.0 wt %). The mixture was shaken for 10 s, and the urea concentration was determined by measuring the absorbance at 422 nm using a UV–vis Lambda 35 spectrophotometer (PerkinElmer, Waltham, MA, USA).

### 2.4 Urea and Phosphorus Release Kinetics in a Vermiculite Column: Simulated Leaching Experiments

Urea and phosphorus release experiments were performed as reported by Ramìrez-Rodrìguez et al. with modifications.^18^ Release was carried out in a vertical column filled with 1 g of sieved vermiculite (particle size <600 μm). This substrate is characterized by a neutral to slightly alkaline pH (7.0–7.5) and a high cation exchange capacity (100–150 mequiv/100 g). Its low intrinsic nutrient content and high water-retention capacity make it suitable for controlled nutrient release studies. Vermiculite was mixed with the materials, and the bottom of the column was blocked with hydrophilic cotton to prevent vermiculite loss. The amount of each material used was selected to keep constant the quantity of urea at 25 mg for each experimental set, and three replicates were performed for each material. A continuous flow of water at a rate of 4.5 mL h^–1^ was applied by using an Ismatec peristaltic pump (Fisher Scientific, Pittsburgh, USA). The water released from the column was collected at five time points: 20, 60, 120, 180, and 240 min. The collected samples were analyzed by ICP–OES to quantify phosphorus and by UV–vis to quantify urea following the method described above. The release behavior of urea from ACP-U and pure urea was further investigated to identify which kinetic model most accurately describes the release pattern. The curves were fitted to the experimental data using a nonlinear least-squares fitting algorithm to obtain the model parameters. Four kinetic models commonly employed in studying urea release behavior were utilized:^19^ (i) zeroth-order kinetic, (ii) first-order kinetic, (iii) Higuchi, and (iv) Korsmeyer-Peppas. The kinetics are described by the following equations:

Zeroth-order model

$$\frac{M_{t}}{M_{\infty }}=k_{0}t$$ 1

First-order model

$$\frac{M_{t}}{M_{\infty }}=1-e^{-k_{1}t}$$ 2

Higuchi model

$$\frac{M_{t}}{M_{\infty }}=k_{H}t^{1/2}$$ 3

Korsmeyer-Peppas model

$$\frac{M_{t}}{M_{\infty }}=k_{KP}t^{n}$$ 4

where *M_t_* and *M*_∞_ represent the remaining mass of urea at time *t* and equilibrium, respectively; *k*_0_ represents the diffusion kinetic constant of the zeroth-order model; *k*_1_ and *k*_H_ are the release constants of the first-order model and Higuchi model, respectively; and *k*_KP_ and *n* are the rate constant and release index of the Korsmeyer-Peppas model, respectively.

In zeroth-order kinetics, the rate of species release is independent of the concentration of the loaded species, and in first-order kinetics, the rate of species release is proportional to the concentration of the species remaining in the dosage form. The Higuchi model is based on the Fickian diffusion model and assumes that the rate-limiting step is the diffusion of species through the matrix. The Korsmeyer-Peppas model is a semiempirical mathematical model commonly used to describe release from polymeric systems. This model is particularly useful for systems exhibiting non-Fickian transport, where both diffusion and advection contribute to the ion release. When *n* = 0.5, the release follows Fickian diffusion (Higuchi kinetic), when 0.5 < *n* < 1, the release is anomalous (non-Fickian), and when *n* = 1, the release follows zeroth-order kinetics.^24^

### 2.5 Greenhouse Experiment

A greenhouse experiment was conducted at the Connecticut Agricultural Experiment Station (CAES, New Haven, CT, USA) from July 2024 to August 2024 with corn (*Zea mays*, hybrid bicolor Sh2 corn (BOLT XR F1) from Johnny’s Selected Seeds, 955 Benton Avenue, Winslow, Maine 04901) using the developed materials as fertilizers. ACP and ACP–U were tested as experimental treatments, while monocalcium phosphate (MCP)—the most common ingredient in commercial phosphorus fertilizers—was used as a control. Each pot contained 500 g of dry soil. The soil used was a sandy loam from the CAES Griswold Research Center (Griswold, CT, USA), with a pH of 6.8, a total phosphorus content of 667 mg kg^1–^, and a bioavailable phosphorus content of 1.47 mg kg^–1^. Urea and KCl were added to balance nitrogen and potassium levels (90 mg of nitrogen and 166 mg of potassium per 1 kg of dry soil). Corn seeds were germinated for 5 days in Petri dishes and then transplanted into pots containing a mixture of 90% soil and 10% vermiculite. Fertilizers were applied to the soil 24 h before transplanting. The experiment included 12 replicate pots per treatment, and plants were grown under greenhouse conditions for 5 weeks and watered from the bottom. Irrigation was performed every other day by placing the pots in water-filled trays. Water was added until the bottom tray (100 mL capacity) was full, ensuring consistent and uniform water availability across treatments. During the experimental period (July–August 2024), plants were exposed to natural sunlight, with a photoperiod ranging from 14 h 56 min to 13 h 28 min and an average greenhouse temperature of approximately 27 °C. On day 35, the relative chlorophyll content was assessed using the single-photon avalanche diode (SPAD) index, which is a widely used indicator of leaf chlorophyll content, closely correlated with biomass production. Measurements were taken with a MultispeQ V2.0 device (PhotosynQ, East Lansing, MI, USA) on the third fully expanded leaf from the top of each maize plant. Plants were then harvested and cleaned with tap water, and the roots and the shoots were weighed separately. To measure plant samples dry biomass, tissues were dried in an oven at 65 °C for 7–10 days until a constant weight was reached. Approximately 100 mg of dried tissue for the ACP-U and MCP treatments was digested with concentrated nitric acid in a hot block (DigiPREP MS, SCP SCIENCE, Quebec City, Canada) at 115 °C for 45 min and diluted with deionized water to a final 2 wt % acid concentration. Macronutrients (Ca, P, Mg, K, and S) in roots and shoots were quantified using ICP–OES (iCAP 6500 Thermo Fisher Scientific, Waltham, MA, USA). Yttrium was used as an internal standard, and a quality control sample was read every 15 samples for calibration verification. The nitrogen content in shoots was assessed with a Leco FP828 nitrogen analyzer (Leco Corporation, Saint Joseph, MI, USA). Approximately 50 mg of ground tissue was analyzed per sample, with lysine used as an internal standard.

Statistical analysis was performed using one-way ANOVA followed by Fisher’s least significant difference (LSD) test to identify significant differences between treatments (*p* < 0.05). This two-phase experiment was designed to compare the effect of fertilizers on plant growth and nutrient retention in soil.

### 2.6 Rhizosphere Microbial Community

At the time of plant harvest, plant root tissue with adhering soil was collected to characterize the rhizosphere bacterial communities. The procedure was performed as described by McPherson et al.^25^ Briefly, rhizosphere soil was removed from root tissues by harvesting ∼10 cm sections of upper roots, followed by placement in a 50 mL falcon tube with 25 mL of sterile saline. The tubes were vortex mixed for 1 min and subsequently centrifuged at 13000 rpm, and the saline solution was decanted. The soil pellet was stored at −80 °C until further DNA processing. Total DNA was extracted from 0.25 g of rhizosphere soil by using the DNeasy PowerSoil kit (Qiagen). PCR amplification of 16S rRNA genes was conducted using the 16S Barcoding Kit (SQK-RAB204; Oxford Nanopore Technologies, Oxford, UK) containing the 27F/1492R primer set and Platinum SuperFi II DNA polymerase (Invitrogen). The PCR reaction also included 7.5 μM mPNA and pPNA peptide nucleic acid (PNA) clamps (mPNA, GGCA­AGTG­TTCT­TCGGA; pPNA, GGCT­CAAC­CCTG­GACAG) to block the amplification of host plant mitochondria and plastid rRNA genes, respectively.^26^ PCR conditions consisted of 94 °C for 2 min followed by 30 cycles of 94 °C for 15 s, 60 °C for 15 s, 68 °C for 15 s, and 4 °C for an infinite hold. The resulting amplification products were verified by gel electrophoresis. The resulting PCR products were mixed at equal molar concentration, and a total of 100 ng of DNA was used for library preparation. MinION sequencing was performed using a R10.4.1 flow cell (Oxford Nanopore Technologies) according to the manufacturer’s instructions. The sequencing was performed for 48 h. Basecalling was performed using Dorado v0.7.1 with superaccurate mode (SUP) models version 4.3 (ONT, dna_r10.4.1_e8.2_400bps_sup@v4.3.0) and version 5.0 (ONT; dna_r10.4.1_e8.2_400bps_sup@v5.0.0). 16S rRNA gene sequences were initially processed using the mothur software package (v. 1.44.2).^27^ Chimeric sequences were identified with the VSEARCH algorithm^28^ as implemented in mothur using the most abundant sequences as a reference for chimera detection. All putative chimeric sequences were removed from the data sets. The 16S rRNA sequences were classified against the SILVA v138 reference database^29^ using the RDP naive Bayesian classifier^30^ as implemented in mothur and sequences identified as belonging to eukaryotes or that were unclassified were removed. The resulting sets of sequences were assigned to amplicon sequence variants, employing a 100% sequence similarity threshold. For analyses of alpha and beta diversity, sequence data sets were analyzed in the phyloseq R package.^31^ The raw data, after demultiplexing, have been submitted to the National Center for Biotechnology Information (NCBI) and can be accessed under project number PRJNA1241238.

## 3 Results and Discussion

### 3.1 Structural and Chemical Characterization of the Samples

The PXRD analysis (Figure 1a) confirms that the ACP sample is amorphous, as indicated by the absence of diffraction peaks. Instead, a broad band centered at around 30° 2θ is observed, which is characteristic of the amorphous phase, as previously reported.^22^ Conversely, the PXRD pattern of the HAP sample indicates its crystalline nature and confirms the presence of a hydroxyapatite phase (JCPDS no. 00-009-0432). The broadness of the diffraction peaks indicates that the material consists of poorly crystalline nanoparticles.

**Figure 1:**
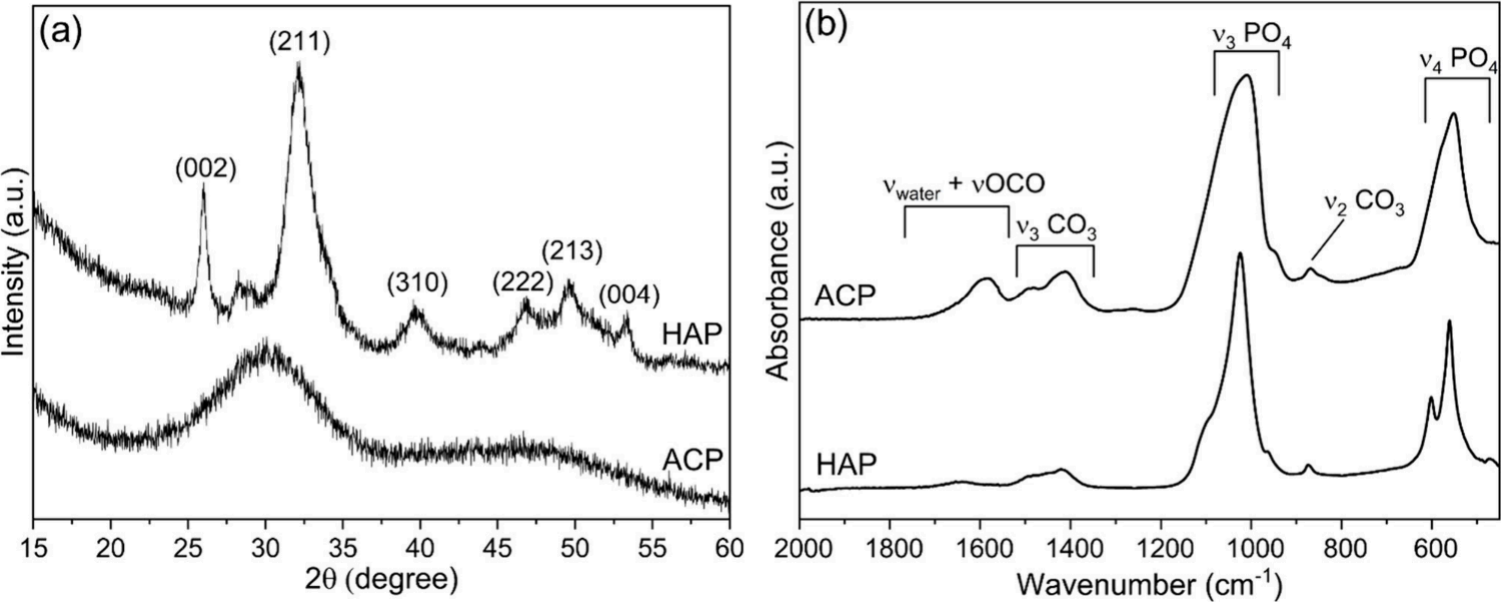
(a) PXRD patterns and (b) FT-IR spectra of ACP and HAP.

The FT-IR spectrum of ACP (Figure 1b) exhibits broad bands characteristic of the amorphous calcium phosphate phase. The absorption bands observed at around 560 and 1050 cm^–1^ correspond to the bending (ν_4_PO_4_) and stretching (ν_3_PO_4_) modes of phosphate groups, respectively.^32^ Additional bands at around 870 cm^–1^ (ν_2_CO_3_) and within the 1400–1500 cm^–1^ range (ν_3_CO_3_) indicate the presence of carbonate ions, while the band near 1560 cm^–1^ (νOCO) is attributed to the asymmetric stretching of −COO^–^ groups from citrate.^32^ The shoulder at around 1600 cm^–1^ is attributed to the bending of water.^29^ These spectral features are consistent with the established characteristics of citrate-stabilized ACP containing carbonate ions. In contrast, the FT-IR spectrum of the HAP sample confirms its crystalline nature, as evidenced by the splitting of the triply degenerate bending mode of apatitic PO_4_ (ν_4_PO_4_) at 605 and 563 cm^–1^ due to the presence of crystalline order.^28^ This sample also displayed a characteristic main band at ca. 1020 cm^–1^ and a shoulder at 1100 cm^–1^ due to the triply degenerate asymmetric stretching mode of the apatitic PO_4_ groups (ν_3_PO_4_). The absence of bands ascribable to the apatitic OH^–^ groups (the vibrational hydroxyl band ν_L_OH at 630 cm^–1^) suggests a low content of hydroxyl due to the low temperature of synthesis as well as to the substitution of the OH groups by CO_3_ as denoted by the occurrence of bands at around 1425 and 875 cm^–1^.

The PXRD patterns of urea-functionalized HAP-U and ACP-U closely resemble those of the pristine materials. Notably, no peaks ascribable to crystalline urea are present in either material, suggesting no formation of a crystalline conglomerate with the calcium phosphate materials. The presence of urea is demonstrated by the FTIR spectra of ACP-U and HAP-U in comparison to that of pure urea (Figure 2b). The spectra display the characteristic absorption bands of urea together with the bands of ACP and HAP previously described. Specifically, the spectrum of urea is characterized by bands at approximately 1672 and 1620 cm^–1^ attributed to the asymmetric δ_as_NH_2_ and symmetric δ_s_NH_2_, respectively, as well as a band at around 1457 cm^–1^ associated with asymmetric stretching of ν_as_C–N and the band at 1560 cm^–1^ attributed to the urea νC=O group.^33–36^

**Figure 2:**
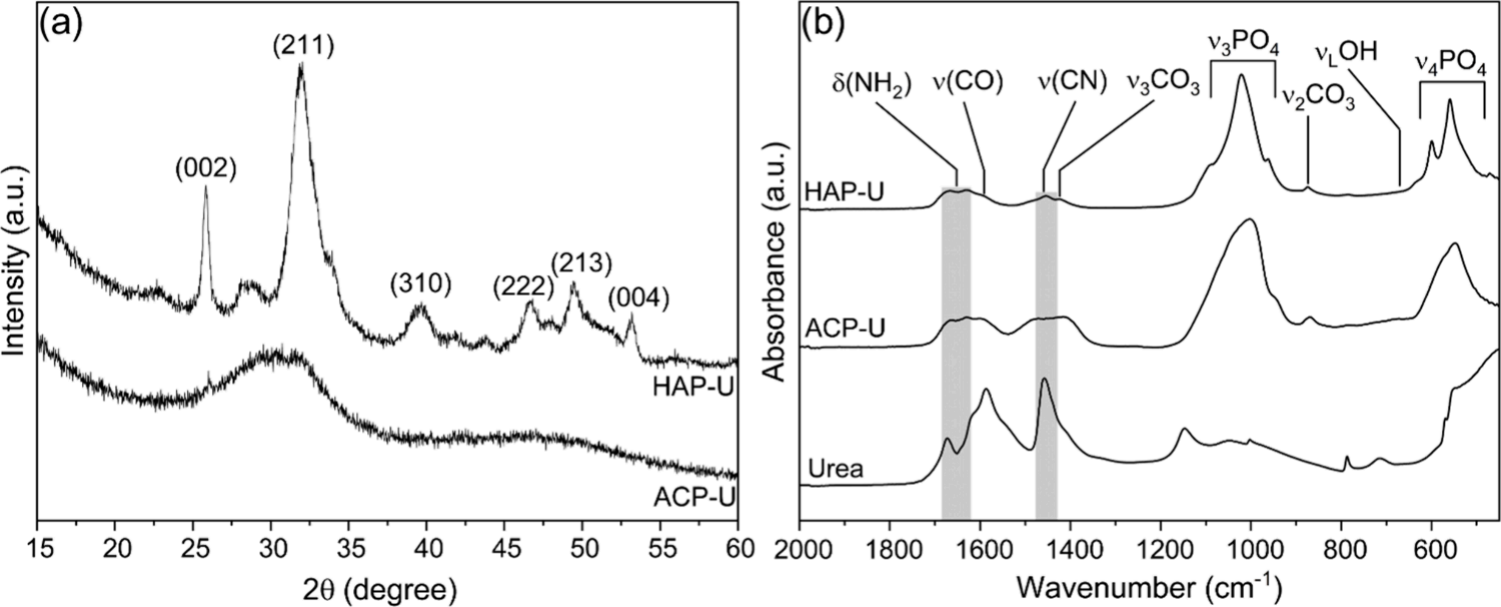
(a) PXRD patterns of ACP-U and HAP-U and (b) FT-IR spectra of urea, HAP-U, and ACP-U.

In the FT-IR spectra of the nanoparticle samples, the urea vibrational bands are superimposed with the bands related to water, carbonate, and citrate. For this reason, the FT-IR spectra of functionalized materials, and as comparison also of nonfunctionalized samples, were deconvoluted into their component peaks to analyze urea chemical bonding and structure. The region between 1300 to 1800 cm^–1^ where the characteristic bands of urea are located was magnified and studied (Figure 3). For HAP (Figure 3a), the deconvolution demonstrated the presence of only carbonate bands at 1476 and 1415 cm^–1^ as well as the water band at 1637 cm^–1^. In the deconvoluted spectrum of ACP (Figure 3b), the analysis revealed bands attributed to citrate at 1583 cm^–1^, water at 1625 cm^–1^, and carbonate at 1491 and 1414 cm^–1^. As expected, no absorption bands characteristic of urea were detected in the spectra of the nonfunctionalized samples.

**Figure 3:**
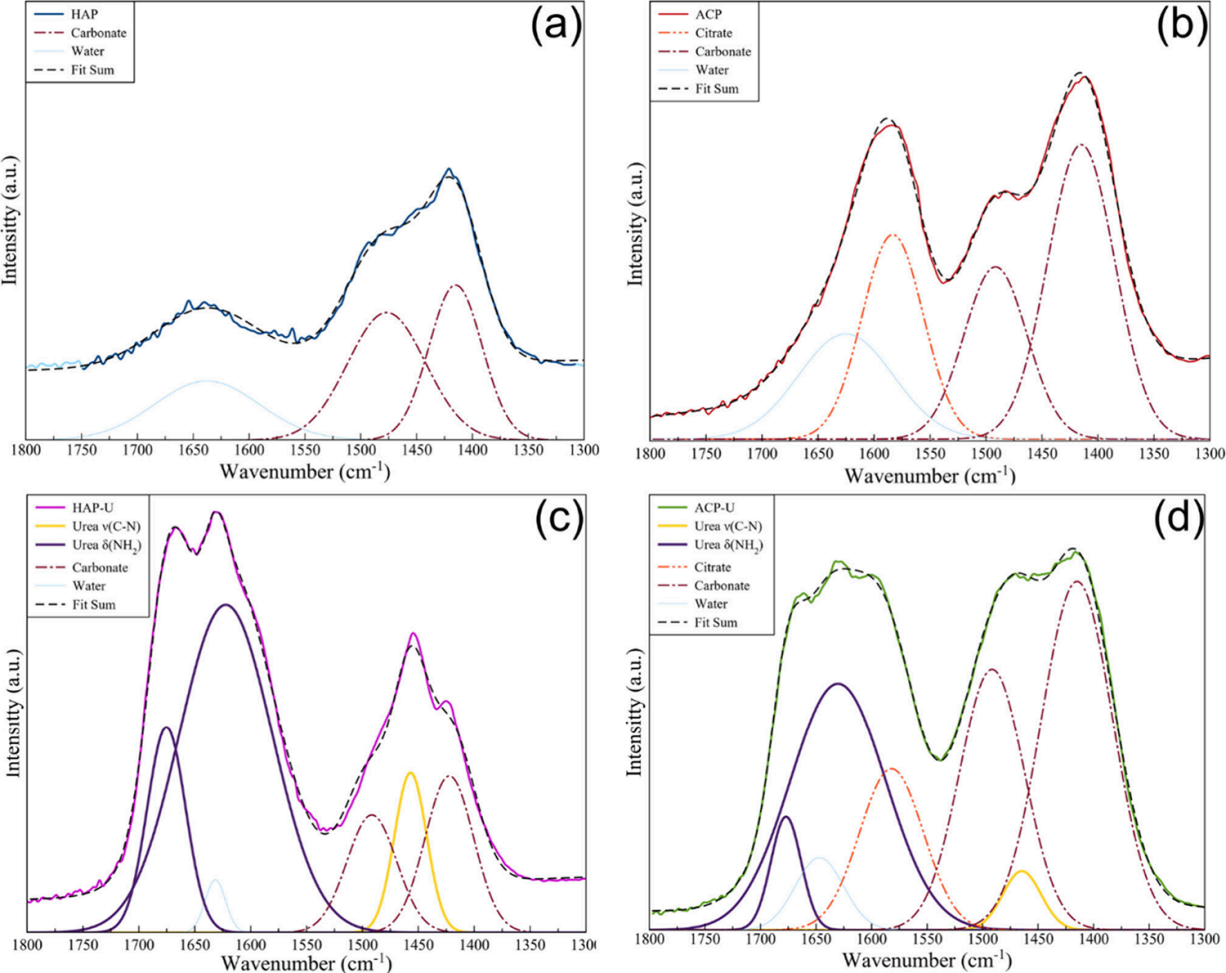
Deconvolution of the FT-IR spectra from 1800 to 1300 cm^–1^ of (a) HAP, (b) ACP, (c) HAP-U, and (d) ACP-U.

The deconvolution analysis of the FTIR spectra of urea-functionalized materials clearly shows the presence of bands associated with urea. For ACP-U (Figure 3c), three characteristic urea bands were identified at 1676, 1630, and 1463 cm^–1^. Similarly, for HAP-U (Figure 3d), the corresponding bands were observed at 1675, 1622, and 1456 cm^–1^. In the spectrum of the HAP-U sample, the urea signals showed only partial shifts compared to those of pure urea (Table 1). In contrast, the bands associated with urea in the spectrum of the ACP-U sample exhibited significant shifts, particularly for the ν_as_C–N and δNH_2_ modes. A prominent shift was observed in the *δ*_as_NH_2_ band that moves from 1620 cm^–1^ in pure urea to 1630 cm^–1^ in ACP-U. Similar shifts in the FT-IR spectra of HAP-U composites have been reported by Fernando et al.^37^ and Channab et al.,^38^ who noted a shift to higher wavenumbers of approximately 10 cm^–1^. Fuan et al.^39^ demonstrated via density functional theory that urea interacts with hydroxyapatite primarily through its amino groups binding to electrophilic Ca^2+^ sites of the hydroxyapatite surface. Our study did not find significant shifts in the HAP-U system but did record a blue shift in the asymmetric and symmetric N–H bending vibrations in the ACP-U system. Since ACP similarly exposes Ca^2+^ sites on its surface,^40^ it is plausible that it interacts with urea through a similar mechanism to HAP. Overall, PXRD and FT-IR results confirmed the surface adsorption of urea on ACP.

**Table 1:** Position of the Urea Peaks Identified in the Spectral Range of 1300–1800 cm^–1^ in the FT-IR Spectra of Urea, HAP-U, and ACP-U

Sample	ν_as_C–N (cm^–1^)	*δ*_a__s_NH_2_ (cm^–1^)	*δ*_s_NH_2_ (cm^–1^)
**HAP-U**	1457	1622	1675
**ACP-U**	1465	1630	1677
**Urea**	1457	1620	1672

The amounts of Ca and P as well as those of N and loaded urea of the samples are reported in Table 2. The Ca/P molar ratio of ACP is 1.64, which is a value higher than the typical Ca/P molar ratio of 1.50 reported for amorphous calcium phosphates precipitated under alkaline conditions.^41^ Previously, we demonstrated that the ACP stabilized by citrate and enriched with carbonate used in this study has a higher Ca/P due to the substitution of carbonate ions for phosphate ions.^32^ The Ca/P ratio of HAP is 1.60, which is typical of poorly crystalline and Ca-deficient hydroxyapatite.^21^ In the HAP-U sample, the Ca/P ratio increased to 1.68. We believe that this increase is due to the faster release of phosphate ions over calcium ions from HAP particles in water during the adsorption of urea. Conversely, ACP-U does not show a significant change in the Ca/P ratio compared to the starting ACP. When comparing the two materials, ACP exhibits a higher urea loading capacity than HAP. This conclusion is supported by both the colorimetric analysis and the CN analyzer measurements, which show consistent results. In fact, assuming that all nitrogen in the materials originates from urea, the nitrogen content of HAP-U and ACP-U corresponds to urea values of 7.2 and 9.9 wt %, respectively, which aligned well with those determined by the colorimetric method. As urea is present as absorbed species to ACP, this finding is in line with the well-documented higher specific surface area of ACP (about 200 m^2^ g^–1^)^22^ compared to that of HAP (about 100 m^2^ g^–1^)^22^ as well as higher reactivity of amorphous nanoparticles compared to that of crystalline materials. These findings are consistent with the greater loading capacity of amorphous calcium phosphate compared to its crystalline counterpart.^18^

**Table 2:** Chemical Compositions of HAP, ACP, HAP-U, and ACP-U^a^

Sample	P (wt %)^a^	Ca (wt %)^a^	Ca/P^a^	Urea (wt %)^b^	N (wt %)^c^
**HAP**	16.4 ± 0.7^a^	33.9 ± 1.4^a^	1.60 ± 0.01^a^		
**ACP**	13.8 ± 0.1^b^	29.2 ± 0.1^b^	1.64 ± 0.01^b^		
**HAP-U**	14.3 ± 0.5^c^	31.2 ± 1.0^b^	1.68 ± 0.01^c^	7.2 ± 0.1^a^	3.36 ± 0.02^a^
**ACP-U**	11.8 ± 1.4^d^	25.1 ± 2.3^c^	1.64 ± 0.01^b^	10.0 ± 0.1^b^	4.65 ± 0.03^b^

a Data are expressed as the mean value ± standard deviation of three replicates. Different letters indicate statistically significant differences among samples for each column (post-hoc Duncan test *p* < 0.05).

b Quantified by ICP–OES.

c Quantified by the UV–vis colorimetric method.

d Quantified by a CN analyzer. a, b, c, and d indicate statistically significant differences between groups (*p* < 0.05).

### 3.2 Urea and Phosphorus Release Kinetics in a Vermiculite Column: Leaching Experiments

The release of urea and phosphorus under leaching conditions was carried out in a vertical bed column (10 cm length and 2 cm diameter) packed with vermiculite (type IV) as a simulated solid medium, mimicking an inert soil (i.e., a low-diffusive medium) (Figure 4a). The quantity of urea in the leachates, reported as a percentage with respect to the total amount of urea in the system, showed that the release of urea from ACP-U was slower compared to that of HAP-U and free urea. Within 60 min, HAP-U and free urea achieved complete release, while ACP-U released only 76 wt % of the initial amount. The complete urea release from ACP-U was achieved after 4 h, with a release rate approximately 6 times slower than that of pure urea. These results demonstrate that ACP-U can effectively delay nitrogen delivery even under continuous and relatively fast water flow (4.5 mL h^–1^), which imposes more severe leaching conditions than those typically used in the literature. The slower initial release of urea (25% reduction) suggests that nitrogen remains available in the substrate for a longer period, potentially improving the NUE.

**Figure 4:**
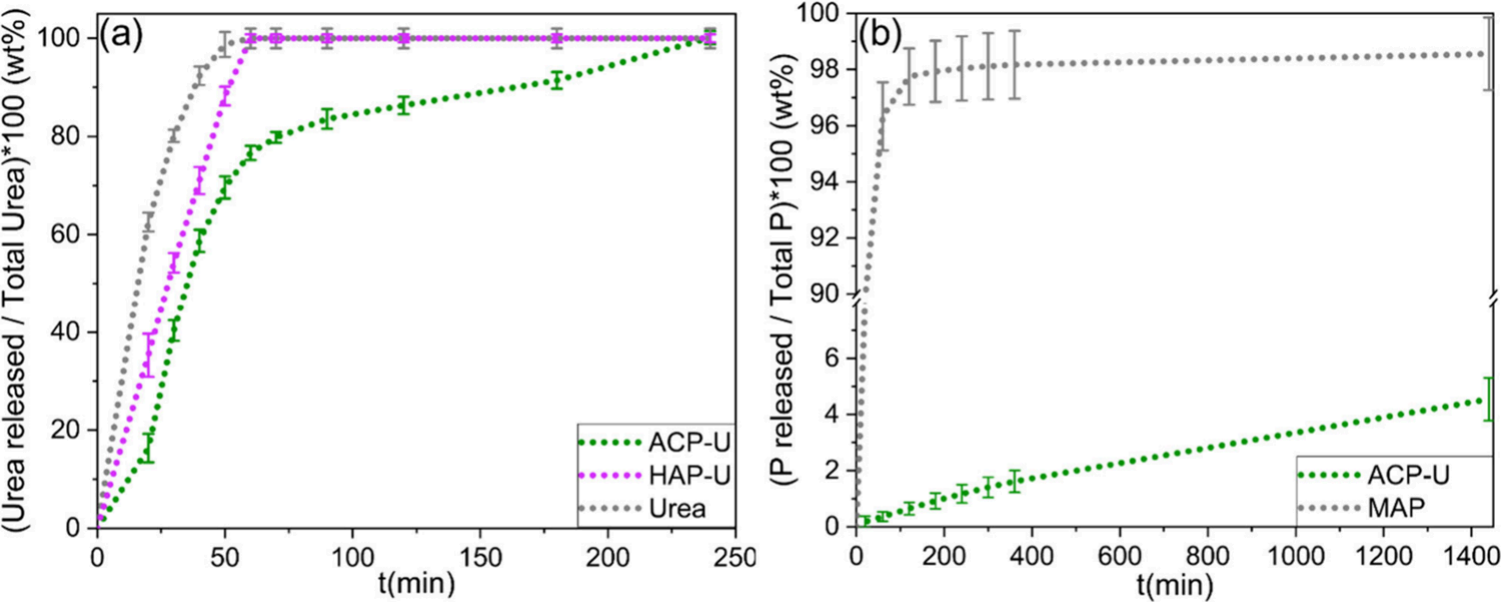
(a) Urea release profiles of ACP-U, HAP-U and free urea. (b) Phosphorus release profiles of ACP-U and MAP. Data are expressed as the mean value ± standard deviation of three replicates.

The results obtained for ACP are comparable with those reported in a study by Carmona et al., who investigated the use of ACP for urea and nitrate delivery.^17^ In their work, the authors conducted a release study in water and observed an initial fast release of 89 wt % of the total nitrogen within the first 15 min, likely due to the weakly bonded urea. The remaining urea was released over 24 h. A similar study was carried out by Ramírez-Rodríguez et al., who examined the release of urea from ACP loaded with urea, synthesized via a one-pot method, in a simulated soil column.^18^ With a flow of 0.86 mL h^–1^, they found that approximately 90% of the urea was released within the first hour, with the remaining fraction being released over 6 h at a rate approximately 10 times slower than that of pure urea.

HAP-U exhibited only a slight reduction in the urea release rate compared to that of pure urea, and this difference was appreciable only in the first minutes of the experiments. At first glance, these results seem to contradict some reports on controlled-release fertilizers based on HAP and urea. For example, Kottegoda et al. reported that pure urea reached complete release in a simulated soil column after 5.3 min, whereas the HAP composite extended the release to 63.6 min. However, our study demonstrated that complete urea release from both materials occurred within 1 h and is consistent with their findings for urea absorbed on HAP. The observed differences in the pure urea release rate can likely be attributed to variations in experimental design across the studies, such as column length, water flow rates, and collection time points, which may have obscured differences.

CaPs are well documented for their ability to slowly release phosphorus, making them highly suitable for controlled-release phosphorus fertilizers.^18^ While HAP is known for its potential to slow urea release, our results demonstrate that ACP nanoparticles are even more effective. Consequently, our experiments on phosphorus release and plant performance focused exclusively on the ACP nanoparticles. To assess the behavior of ACP-U to release phosphorus, we quantified the amount of phosphorus in the leachates collected in the previous experiment. ACP-U showed a significantly extended phosphorus release profile compared to monoammonium phosphate (MAP), used here as a model for conventional phosphorus fertilizers. The amount of MAP used for this experiment was adjusted to match the total phosphate content of ACP-U. As illustrated in Figure 4b, MAP released the entirety of its phosphorus content within 2 h during the leaching experiment. In contrast, ACP-U released only 4 wt % of its total phosphorus in 48 h. While these initial leaching tests were performed using vermiculite to ensure a homogeneous and controlled matrix, we acknowledge that real soil conditions can influence nutrient release behavior. The reported results show that ACP-U nanoparticles have a higher ability to retain nutrients, slowing the release of both urea and phosphorus.

Figure 5 illustrates the fitting of the urea release kinetics of ACP-U and pure urea according to four mathematical models commonly reported in the literature (eqs 1–4).^20^ The release kinetic parameters of different models are shown in Table 3.

**Figure 5:**
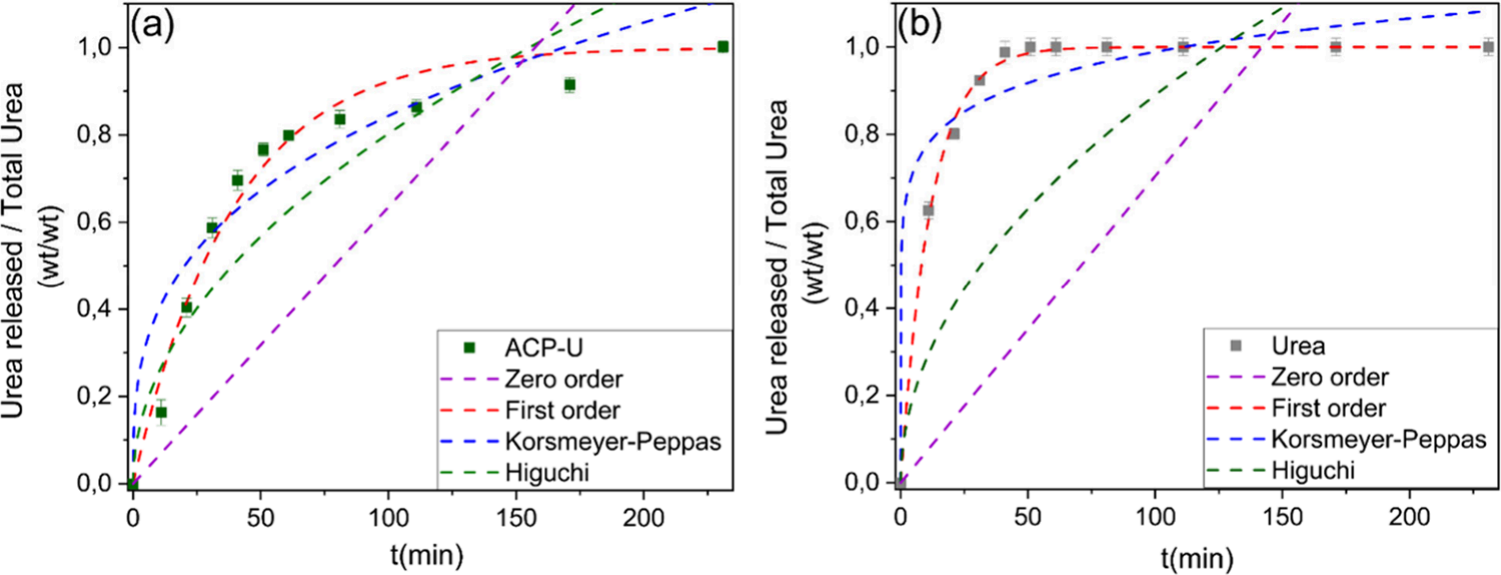
Fitting analysis of the urea kinetic release profile of (a) ACP-U and (b) free urea. Data points represent the mean values ± standard deviation from *n* = 3 independent replicates. Dotted lines indicate fits of the kinetic models.

**Table 3:** Parameters of the Kinetic Models Used for the Urea Release of ACP-U and Free Urea

Model	Parameters	ACP-U	Free urea
Zeroth order	*R* ^2^	0.705	0.705
	*k*_0_	0.006 ± (4.3 × 10^–5^)	0.007 ± (4.8 × 10^–5^)
First order	*R* ^2^	0.976	0.998
	*k*_1_	0.026 ± 0.001	0.085 ± 0.002
Higuchi	*R* ^2^	0.865	0.580
	*k*_H_	0.080 ± (2.7 × 10^–4^)	0.0890 ± (7.1 × 10^–4^)
Korsmeyer-Peppas	*R* ^2^	0.899	0.940
	*k*_KP_	0.188 ± 0.004	0.602 ± 0.078
	*n*	0.326 ± 0.005	0.108 ± 0.030

These models provide valuable insight into the dominant release mechanisms of the tested materials. The results on pure urea agree with those obtained by Zhang et al*.*, who found that *R* ^2^ values for this material were high for first-order and Korsmeyer-Peppas models and were much lower for zeroth-order and Higuchi kinetic models.^42^

Ramírez-Rodríguez et al.^18^ employed ACP-U nanoparticles synthesized by a one-pot precipitation method and assessed the release of urea from pressed powders in the form of tablets. Similarly to their study, we found a good correlation with the first-order kinetic approach to modeling the release of urea for both crystalline urea and ACP-U, with high *R* ^2^ values, indicating a strong correlation between the model and experimental data. Furthermore, the release rate constant (*K*), a key parameter for quantifying the rate of urea release, was significantly lower for ACP-U than for pure urea.

The Higuchi model partially fits the release of urea from ACP-U. This model is typically associated with release systems where diffusion within the matrix is the rate-limiting step, which could describe the diffusion of urea molecules through ACP. However, despite the relatively good fitting value, the shape of the Higuchi curve (Figure 5a) does not align with the experimental data.

Both the Korsmeyer-Peppas and first-order models gave high *R* ^2^ values. Good fitting values for both models can be found in a work on urea loaded into a biochar pellet.^42^ In the literature, the Korsmeyer–Peppas model has often provided good fits for urea release from polymers such as alginate and poly­(vinyl alcohol).^43,44^ However, it is unlikely that materials as diverse as porous biochar (with a high SSA), organic polymers, and ACP share the same release mechanisms of urea. Moreover, in our study the Korsmeyer–Peppas model yielded an unusually high *k* value and a very low *n*, effectively minimizing the sum of squared residuals at the expense of physical interpretability. This suggests that the model is overfitting the data rather than capturing the true underlying release mechanism for ACP-U. Consequently, we believe that overall a first-order model provides a more reliable description of urea release from ACP, which is a system where the urea release process is dominated by its dissolution rather than by diffusion within the matrix.^45^

### 3.3 Greenhouse Experiment

A greenhouse experiment was carried out to test the biomass yield and macronutrient profile of corn (*Zea mays*) treated with ACP-based fertilizers. The results demonstrate a significant improvement in biomass yield when using ACP-U. The dry weight of corn plants (Figure 6a) treated with this sample reached an average of 4.02 ± 1.89 g, which corresponds to a 160% increase of dry biomass compared to ACP (1.55 ± 0.65 g) and a 100% increase in comparison to the MCP control (2.04 ± 0.84 g). Conversely, there was no statistically significant difference between ACP and the MCP control. It is important to remark that free urea was included in the samples of ACP and MCP to balance the quantity of nitrogen in all of the treatments. The statistically significant increase of SPAD values in ACP-U-treated plants (Figure 6b) suggests that this material promotes chlorophyll biosynthesis and optimizes photosynthetic performance, thus increasing biomass. This assumption is further supported by a significant increase in Mg concentration (Figure 7a,b) in tissues for ACP-U-treated plants compared to the control, given that Mg is a fundamental component of chlorophyll.

**Figure 6:**
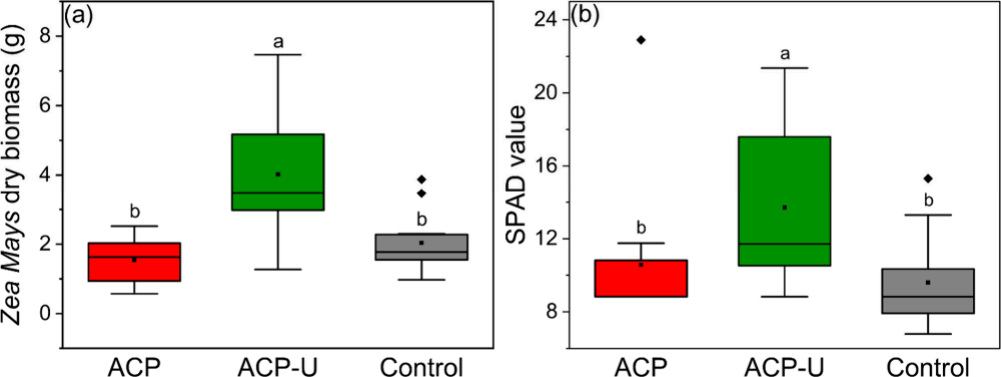
(a) Total dry weight per plant and (b) SPAD value of corn (*Zea mays*) under different treatments: ACP, ACP-U, and Control (MCP). Columns labeled with different letters indicate statistically significant differences at *p* < 0.05, as determined by one-way ANOVA followed by Fisher’s LSD test. Outliers are marked with ◆, the mean with ■, and the median with ―. The interquartile range (IQR) is represented within a 1.5 × IQR interval. The experiment included 12 replicate pots per treatment.

**Figure 7:**
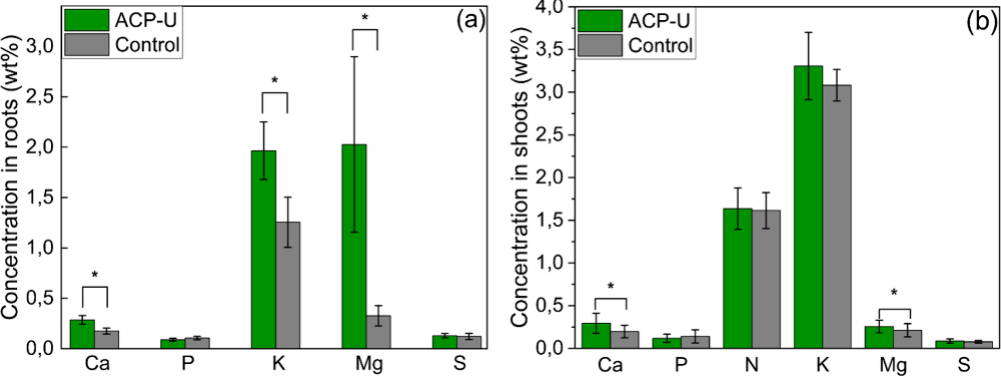
Macronutrient concentrations in (a) roots and (b) shoots of corn (*Zea mays*) under ACP-U and Control (MCP) treatments. Elements showing significant differences (*p* < 0.05) between ACP-U and the control are marked with an asterisk (*), as determined by one-way ANOVA followed by Fisher’s LSD test. Data are shown as the mean ± standard deviation (*n* = 12 replicates).

Similarly, the Ca concentration in plant tissues increases as this element plays a crucial role in plant growth by acting as a counterion for organic and inorganic ions in the vacuole and facilitating intracellular communication through cytosolic Ca^2+^ signaling. We also observed a higher concentration of K in roots, although not in shoots. However, no significant differences were observed in P and S concentrations in either shoots or roots or in N concentration in shoots (Figure 7b). The overall enhancement in nutrient content, particularly Ca, Mg, and K, likely contributed to the increased biomass.

Nitrogen concentration in the roots was not reported due to the limited amount of root material available from all replicates, which prevents reliable analysis. Additionally, the ACP treatment was excluded from the nutrient concentration figure because, in previous measurements (biomass and SPAD index, Figure 6), it showed no statistically significant differences from the control. Therefore, we focused on treatments that presented clear physiological responses.

It is important to underline that the performance of ACP-based fertilizers is strongly influenced by soil chemistry, particularly pH and the presence of calcium, aluminum, and iron. In acidic soils, ACP particles can dissolve faster, releasing phosphate and calcium ions more rapidly over time. Nitrogen dynamics are also affected by soil pH: ammonia volatilization is favored under alkaline conditions, while acidic environments suppress nitrification and may lead to ammonium accumulation.^46^ Additionally, phosphate ions can be rapidly immobilized by Al and Fe oxides, reducing their biovailability.^47^ Future work should assess the performance of ACP-based fertilizers across a broader range of soil pH and variable Al/Fe content.

### 3.4 Soil Microbial Community

An analysis of the rhizosphere bacterial community diversity revealed a notable and consistent pattern of homogeneity across all treatment groups. The assessment of alpha diversity, using both ASV richness and the Shannon diversity index, indicated no discernible impact of treatments on the number or evenness of bacterial taxa (Figure 8a,b). Furthermore, beta diversity, which examines variations in community composition between samples, showed no significant differences between treatments, as visualized by principal coordinate analysis (PCoA) and nonmetric multidimensional scaling (NMDS) based on Bray–Curtis dissimilarity (Figure 8c,d). These results indicate that the overall structure and composition of the rhizosphere bacterial communities remained stable across the different fertilization treatments.

**Figure 8:**
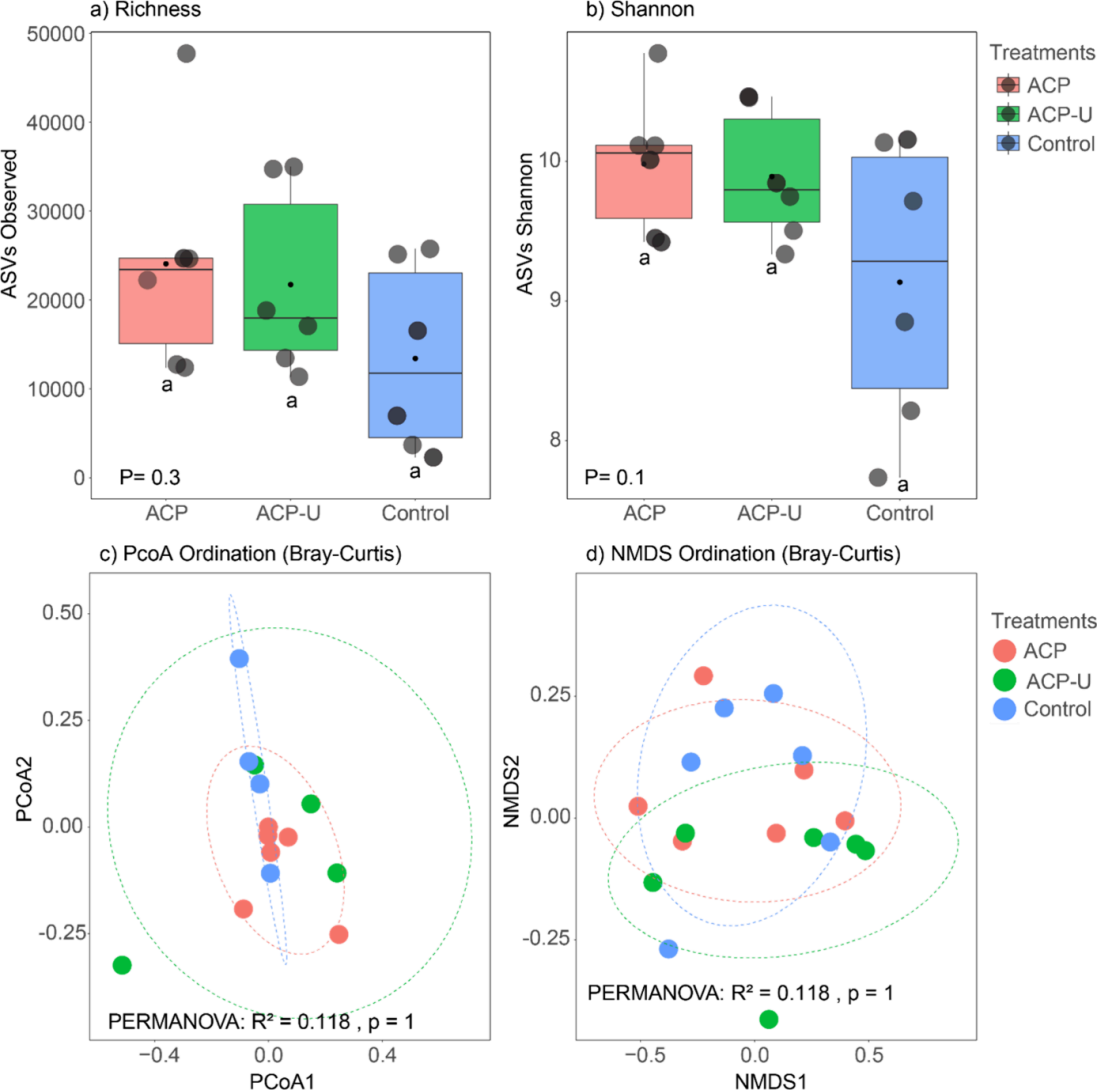
Diversity of rhizosphere bacterial communities. Alpha diversity is represented by (a) ASV richness (number of recovered amplicon sequence variants) and (b) the Shannon diversity index. Analysis of variance (ANOVA) revealed no significant differences in alpha diversity between treatment groups. Beta diversity was assessed using (c) principal coordinate analysis (PCoA) and (d) nonmetric multidimensional scaling (NMDS) based on Bray–Curtis dissimilarity. Permutational multivariate analysis of variance (PERMANOVA) revealed no significant differences in community structure between treatment groups.

We additionally investigated the taxonomic composition of the bacterial communities and observed a high level of community consistency among samples, with 76% of bacterial families and 70% of genera being conserved among all three fertilization treatments (Figure 9).

**Figure 9:**
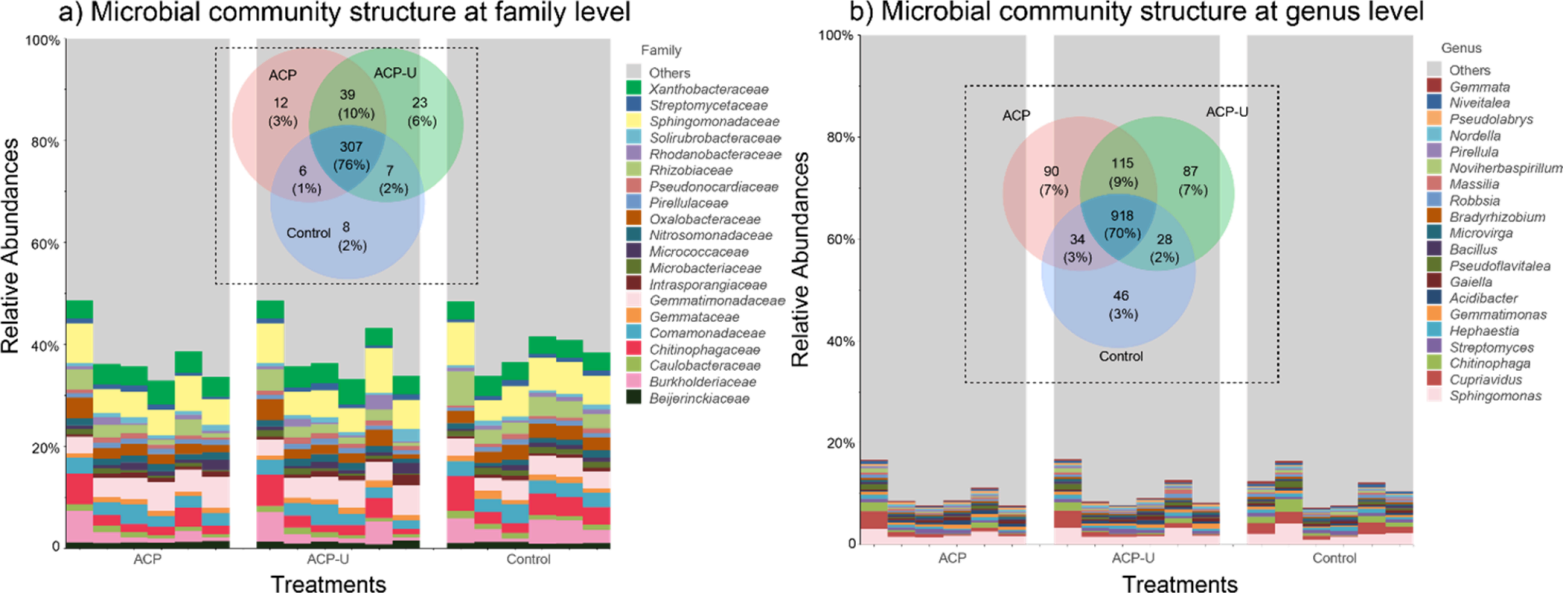
(a) Microbial community structure at the family level and (b) microbial community structure at the genus level for ACP, ACP-U, and Control (MCP) treatments. Stacked bar plots showing the relative abundance of microbial communities at the (a) family and (b) genus levels across different treatments. Each treatment includes six columns representing biological replicates, with each column corresponding to one sample. Relative abundances were calculated based on 16S rRNA gene sequencing data and normalized within each sample. The plots display the top 20 most abundant families and genera, respectively, with all remaining taxa grouped under “Others.” Each panel also includes a Venn diagram showing the number and percentage of families (a) and genera (b) that are unique to or shared among treatments. All plots were generated in R (version 4.4.3) using the “ggplot2” and “VennDiagram” packages.

Taken together, our findings suggest that the observed differences in plant growth phenotypes are not a consequence of substantial alterations in the overall structure of the rhizosphere bacterial communities.

Overall, this study provides evidence that ACP-U nanoparticles may enhance nutrient retention and plant performance. Structural and chemical characterization confirmed a higher urea loading capacity for ACP compared with HAP, likely due to its greater surface area and reactivity. Leaching experiments using a simulated soil column revealed that ACP-U exhibited a slower release of both urea and phosphorus with respect to the controls, suggesting an improved nutrient retention. Greenhouse trials under controlled conditions showed trends of enhanced biomass, chlorophyll content, and macronutrient concentrations in ACP-U-treated corn plants. While these results are encouraging, further validation is needed to confirm the consistency and robustness of the observed effects. Future research should include long-term studies under real soil and climatic conditions to evaluate the agronomic relevance and environmental impact of ACP-U on a larger scale. Moreover, since nutrient requirements vary throughout the plant growth cycle and differ among species, additional studies are necessary to optimize the NUE of the developed materials. In particular, testing the nanofertilizer during the vegetative growth stage, when nitrogen uptake rates are highest, could help refine application strategies.
